# New Means of Canine Leishmaniasis Transmission in North America: The Possibility of Transmission to Humans Still Unknown

**DOI:** 10.1155/2009/802712

**Published:** 2009-06-04

**Authors:** Christine A. Petersen

**Affiliations:** 2764 Veterinary Medicine, Department of Veterinary Pathology, Iowa State University, Ames, IA 50011, USA

## Abstract

At present it is not possible to determine in advance the outcome of *Leishmania infantum* infection. Canine Visceral Leishmaniasis (VL), caused by *Le. infantum*, is a natural disease process which offers a insight into the interaction of the host and resultant disease outcome. Canine VL results in the same altered pathophysiology and immunodysregulation seen in humans. VL in US dogs is likely to be transmitted primarily via nontraditional, nonvector means. VL mediated by *Le. infantum* is endemic in U.S. Foxhound dogs, with vertical transmission likely to be the novel primary means of transmission. This population of dogs offers an opportunity to identify host factors of natural disease. Prevention of human clinical visceral leishmaniasis can occur only by better understanding the disease ecology of the primary reservoir host: the dog.

## 1. Introduction

**Figure 1:**
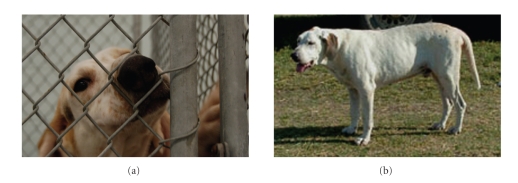
Both humans and dogs have a wide clinical presentation with *Le. infantum*. These presentations vary from (a) no clinical signs and robust healthy behavior in a young healthy Foxhound to (b) multiple clinical signs (polysymptomatic) including poor hair coat, enlarged liver and spleen, and crusty cutaneous lesions seen on the rump in an older hound.

*Leishmania infantum* is the causative agent of visceral leishmaniasis (VL) in the Mediterranean Basin and more recently North America. Natural hosts include dogs and humans [1], and transmission is usually via a sand fly vector. Infected dogs are the primary reservoir for zoonotic visceral leishmaniasis in endemic regions and are the most significant risk factor predisposing humans to infection [2]. Both dogs and humans have a wide range of clinical presentation due to infection with *Le. infantum*, ranging from asymptomatic to fatal visceralizing disease (Figure 1). Host factors which determine clinical outcome are poorly understood. When clinical symptoms (signs) in both humans and dogs occur, they include enlarged lymph nodes and hepato- and splenomegaly due to parasitic invasion of the reticulo-endothelial system of phagocytic lymphocytes [3]. Visceral leishmaniasis symptoms persist in both humans and canine patients for several weeks to months before patients seek medical care. In the meanwhile these patients are at risk of death from bacterial coinfections, massive bleeding, severe anemia [3], or renal failure in veterinary patients. A better understanding of this neglected disease, particularly the host and vector factors which lead to disease transmission and/or predict clinical outcome, is needed to optimally prevent clinical disease outright but otherwise bring these patients to medical attention faster, have them diagnosed correctly, and treated successfully.

## 2. Transmission of *Le. infantum*

Dogs are the primary mammalian reservoir for *Le. infantum* infection in endemic regions and are the most significant risk factor predisposing humans to infection [2]. In endemic areas, the primary means of transmission is vector-borne via the sand fly. Canine infection in the US suggests a possible human health threat if domestic sand fly species are capable of *Leishmania* transmission. Vector-borne transmission has not been shown in the US to date [4, 5]. Vertical transmission appears to be a major means of transmission in Foxhounds in the US [4]. The frequency of vertical transmission in endemic areas is unknown due to the overwhelming likelihood of vector contact [6]. 

A potential sand fly vector of *Le. infantum*, *Lutzomyia shannoni*, is present within Southern and Southeastern United States [4]. *Lu. shannoni* is known to bite dogs and other mammals and has been incriminated in the transmission of *Le. brasiliensis* in South and Central America [7]. Anecdotal data indicate that US species of *Lu. shannoni* can become infected with *Le. infantum*, but it is not known whether these flies permit *Le. infantum* development into infectious metacyclic parasites. Vector feeding preferences can importantly influence disease transmission. In the US, *Lu. shannoni* has also been shown to bite dogs (Rowton personal communication). Although vector feeding preferences can significantly influence disease transmission, host preference for *Lu. shannoni* in the US is currently not known.

## 3. Sand Fly Preference for Canines as Food Source

In South and Central America, several investigators have demonstrated that dogs are an important blood source to the principal vector of visceral leishmaniasis: *Lu. longipalpis * [8, 9]. Data from a study of the emergence of visceral leishmaniasis in Central Israel in the mid 1990's [10] suggested that a high prevalence of infected dogs (11.5%) contributed to the onset of the disease in humans. In Brazil, *Lu. longipalpis* is frequently found in houses with dogs [11]. A recent study detected high levels of antisand fly saliva antibodies in dogs from an endemic area of transmission of *Le. infantum* in Brazil compared to dogs from nonendemic areas [12], suggesting high exposure to the visceral leishmaniasis vector *Lu. longipalpis*. Dogs play a significant role in the spread and maintenance of *Le. infantum* in endemic areas, although it is not completely clear why dogs are more attractive as a blood source to sand flies. Using the *host selectivity index*, defined as the number of sand flies that feed on a given host relative to the available biomass of the host [13], *Lu*. *pseudolongipalpis*, a vector of visceral leishmaniasis in Colombia, preferred dogs as blood source. In contrast, *Lu. longipalpis* was shown to have no particular preference for specific vertebrate hosts and to be opportunistic feeders [14–17]. There has only been one substantial study looking at the feeding preferences of *Lu. shannoni* in the US [18]. This study was performed on a largely uninhabited barrier island off Georgia and did not find dogs to be a primary food source, but this was most likely because there were very few dogs present on the island. In many settings dogs have been shown to be a link between sylvatic and domestic cycles of visceral leishmaniasis. Dogs often cross forest-edge boundaries, thereby potentially bringing parasites to, or from, sylvatic systems and to and from other potential mammal hosts (such as foxes and opossums). In the US, due to frequent exchange of Foxhounds between kennels and these dogs' penchant for spending time in the woods, Foxhounds may be a primary focal point for transmission of *Le. infantum* to sand flies. Thus, if *Lu. shannoni* indeed prefers to feed on dogs in comparison to other mammals, infected dogs are more likely than other mammals to serve as a source of *Le. infantum* to an uninfected fly. It is important to determine whether *Lu. shannoni* feeds on dogs and further if US sand flies are permissive to parasite development into infectious metacyclic promastigotes.

## 4. Visceral Leishmaniasis in Foxhounds

Visceral Leishmaniasis is classically transmitted to a suitable mammalian host by the bite of an infected sand fly after which the promastigote form of the parasite is phagocytosed by macrophages [1]. Although endemic in many parts of the world, this disease has only recently been described as transmitted solely within the US [19]. Previously, sporadic cases have been reported in the United States, in human and canine travelers returning to the US from endemic areas [5]. However, in 2000, a kennel in New York State reported four Foxhounds with no travel history to be infected with *Le. Infantum *[19]. By 2005, 60 kennels in 22 states and two Canadian provinces had reported seropositive Foxhounds [20]. Nonvector-based mechanisms postulated for transmission of canine visceral leishmaniasis in the US include vertical transmission (transplacental or transmammary) and horizontal transmission by direct contact with infected cells in blood [4, 5, 21]. Transmission has been documented via packed red blood cell transfusion from infected Foxhounds [22]. It is not known how frequently vertical transmission occurs naturally in endemic areas. There are reports of congenital transmission of visceral leishmaniasis in humans and during experimental *Leishmania* infection of beagles [20]. Pathology of visceral leishmaniasis of US Foxhounds was equivalent to that seen in dogs and humans infected in endemic areas through sand fly transmission [21]. Whether vertical transmission itself is solely responsible for the focus of disease in this breed of dogs or whether there are genetic factors predisposing particular lineages of Foxhounds should be further investigated.

## 5. Diagnosis of Visceral Leishmaniasis

In both humans and dogs, infection with *Leishmania infantum* frequently does not equate with clinical illness. The ratio of incident asymptomatic infection to incident clinical cases varies with location, vector and parasite. Ratios of 18:1 in Brazil and 50:1 in Spain have been observed in human populations [3] and is estimated to be 2:1 in high-risk US Foxhounds. Different means of transmission, as observed in US Foxhounds, will also alter this ratio. At present, diagnosis and control of visceral leishmaniasis is difficult as both humans and dogs can be infected but seronegative for years [23]. Various means of serology are the primary diagnostic tests used for surveillance of visceral leishmaniasis. For public health surveillance in the US where this disease is not endemic in humans, testing is performed via an indirect fluorescent antibody assay (IFA) by the Centers for Disease Control and Prevention (CDC). IFA is sufficient for screening purposes, but is found to cross react with antibodies to the kinetoplastid *Trypanosoma cruzi*. *T. cruzi* infects dogs in the Southeastern US, thus further testing is required to determine parasite specificity unless clinical signs are much more consistent with one infection over the other; for example, cardiomyopathy in the case of Chagas' disease. Other serologic tests are available in the US for detection of canine leishmaniasis including a highly sensitive and specifc kELISA available through the Cornell University diagnostic laboratory and a K39-antigen based assay available through Heska. Positive serology in Foxhounds appears to more closely correlate with the appearance of clinical disease than incidence of infection. Reports have shown that qPCR performed by a well-regulated and stringently tested laboratory can be a more sensitive test for *Le. infantum* infection in dogs and can detect asymptomatic dogs and/or dogs that have not yet to seroconvert [21]. qPCR is available through Iowa State University and the CDC.

## 6. IL-10 and Pathogenesis of VL

Mechanisms underlying systemic spread of *Leishmania infantum *during VL are not well understood. Mammalian host responses which prevent progression to clinical VL has been shown to be dependent on promoting T helper-1 IFN-*γ* production-based immunity and parasiticidal activity within infected macrophages [3]. A key immunological feature of late stage clinical VL in either humans or dogs is an inability to proliferate or to produce IFN-*γ* in response to *Leishmania* antigen ([24] and Petersen preliminary data). Pharmacologically-cured individuals are resistant to re-infection and mount antigen-specific IFN-*γ* responses in vitro, indicating that there is not an inherent defect in host CD4+ T cell responses of clinical patients once they have reached this stage. The actual factors which influence clinically-observed infection with *Le. infantum* are still mainly speculative [25]. One study of genetic factors predisposing to clinical VL identified a TNF-*α* allele associated with high serum levels of this cytokine [26]. High levels of TNF-*α* have been proposed to stimulate production of regulatory cytokines, specifically IL-10, as a homeostatic response to prevent further inflammation-mediated pathology. High leisonal IL-10 mRNA production is frequently found in human patients with VL [25, 27], and produced by polysymptomatic Foxhounds (Petersen preliminary data). IL-10 can be produced by many cell types including T cells, B cells and macrophages. One of the proposed mechanisms of IL-10 promotion of VL is by conditioning macrophages for parasite growth and survival versus killing. The best means of determining how leisonal IL-10 contributes to disease outcome would *ex vivo* study of splenic cells [27], which is not possible in human patients.

## 7. Genetic Factors Related to Visceral Leishmaniasis Disease Susceptibility

Although several genetic polymorphisms, including alterations in TNF-*α* and solute carrier family 11A1 (SLC11A1, formerly NRAMP1) allelic expression, have been indicated to predispose to disease [26, 28], causative factors of disease susceptibility in both humans and dogs, specifically those associated with heritability, remain elusive. Breed type has also been shown to alter the response to therapy, suggesting that canine breed-related genetic factors modulate disease progression and are therefore prognostically significant [29]. 

Numerous Foxhounds have tested positive for visceral leishmaniasis in the US and infection appears to be endemic only within this breed in the US If vertical transmission is indeed the primary route of transmission in these dogs, a particular genetic susceptibility is not absolutely necessary for widespread infection to occur in the Foxhound population. Both the observance of visceral leishmaniasis within specific families of Foxhounds and finding hounds that are *Leishmania* disease-resistant suggests that it is highly likely that particular genetic traits of dogs at least in part determine which dogs develop visceral leishmaniasis versus remain clinically disease-free.

## 8. Treatment/Prognosis

Treatment of visceral leishmaniasis (VL) is rarely 100% curative. Prognosis for emaciated chronically infected dogs is very poor and in these cases euthanasia should be considered. Due to difficulty obtaining certain drugs in the United States, treatment in dogs is recommended to begin with allopurinol (Zyloric). This drug is efficacious and relatively nontoxic when used as a maintenance drug. Clinical remission is often achieved when used alone. Relapses are common when treatment ceases, complete cures are rare but survival occurs in 80% of cases over 4 years if renal insufficiency is not present when treatment is initiated. This drug is sometimes used in combination both in dogs and humans with pentavalent antimony (Glucantime), as drug resistance is seen for pentavalent antimony alone in endemic areas (France, Spain, and Italy). Pentavalent antimonials are not licensed for use in the United States and can only be obtained via an investigational drug use protocol from the CDC [30, 31]. The two main drugs in this class are (1) sodium stibogluconate (Pentostam, Wellcome Foundation Ltd, UK), which requires daily injection and has severe side effects, and (2) meglutamine antimoniate (Glucantime, Pfizer/Merial, France), which has less side effects. Amphotericin B in the lipid emulsion or liposomal form is relatively nonnephrotoxic and is effective against the organism, although it is not thought to be superior to allopurinol as it is still both more costly and more toxic. Renal insufficiency must be treated prior to giving antimonial drugs or amphotericin B as prognosis is dependent on renal function at the onset of treatment. Treatment efficacy is best monitored by clinical improvement and presence of organisms in biopsy or as measured by rigorously controlled qPCR. Relapses occur a few months to a year after therapy, so dogs should be rechecked at least every 2 months after the end of treatment. Prognosis for a cure is very guarded, but therapy does provide infected dogs improved quality of life.

Second-line drugs, which require further clinical studies to understand their efficacy in both dogs and humans, include miltefosine (Impavido or Miltex) and paromomycin (Humantin). Paromymycin has been shown to have fewer side effects than other drugs in humans. Use of this drug has been primarily targeted to the cutaneous versions of *Leishmania*, less is known about its ability to remove organ-based infection. There is no effective vaccine against CVL available in the United States. A secreted parasite antigen-based vaccine has recently been licensed for use in dogs in Brazil. Sand fly vector control measures, including deltamethrin or permethrin-impregnated collars are useful to date to prevent disease [32]. In many countries, due to the tie of canine infection to human disease, culling of dogs is still used as a means to prevent human disease [33, 34].

## 9. Summary

Factors which contribute to clinical VL are poorly understood. Canine VL mimics both immunology and pathophysiology of human disease. *Leishmania infantum* infection is endemic in the US Foxhound population. Study of these naturally infected dogs allows insight into the mechanism of lesional IL-10 and other host factors in promoting clinical disease. A cohort population of domestic dogs, specifically American Foxhounds, provides a unique and valuable model system to define vector and host factors that mediate presentation with clinical visceral leishmaniasis. Current evidence indicates that nontraditional means of transmission, particularly vertical transmission, may be a primary route of transmission of the parasite in US dogs, although *Lutzomyia* species in the US may be involved in transmission. Further study is necessary to better understand the impact of vertical transmission of leishmaniasis on the persistence of this disease and determine the likelihood of vector-borne transmission in the US.
